# Impact of health system engagement on the health and well-being of people who use drugs: a realist review protocol

**DOI:** 10.1186/s13643-022-01938-z

**Published:** 2022-04-13

**Authors:** Ginetta Salvalaggio, Lawrence Ferguson, Hannah L. Brooks, Sandra Campbell, Vanessa Gladue, Elaine Hyshka, Linda Lam, Heather Morris, Lara Nixon, Jane Springett

**Affiliations:** 1 Department of Family Medicine, University of Alberta, 6-10 University Terrace, Edmonton, AB T6G 2T4 Canada; 2 Faculty of Medicine & Dentistry, University of Alberta, 8440 112 St. NW, Edmonton, AB T6G 2R7 Canada; 3 School of Public Health, University of Alberta, 3-300 Edmonton Clinic Health Academy, 11405-87 Ave. NW, Edmonton, AB T6G 1C9 Canada; 4 John W. Scott Health Sciences Library, University of Alberta, 2K3.28 Mackenzie Centre, Edmonton, AB T6G 2R7 Canada; 5 Alberta Alliance Who Educate and Advocate Responsibly, 10116 105 Ave. NW, Edmonton, AB T5H 0K2 Canada; 6 Department of Family Medicine, University of Calgary, G012 Health Sciences Centre, 3330 Hospital Dr NW, Calgary, AB T2N 4N1 Canada; 7 Centre for Healthy Communities, School of Public Health, University of Alberta, 3-289 Edmonton Clinic Health Academy 11405-87 Ave. NW, Edmonton, AB T6G 1C9 Canada

**Keywords:** Realist review, Realist synthesis, People who use drugs, Community engagement, Health system engagement

## Abstract

**Background:**

Although community-level benefits of health system engagement (i.e., health service planning, delivery, and quality improvement, engaged research and evaluation, and collaborative advocacy) are well established, individual-level impacts on the health and well-being of community members are less explored, in particular for people who use or have used illegal drugs (PWUD). Capacity building, personal growth, reduced/safer drug use, and other positive outcomes may or may not be experienced by PWUD involved in engagement activities. Indeed, PWUD may also encounter stigma and harm when interacting with healthcare and academic structures. Our objective is to uncover why, how, and under what circumstances positive and negative health outcomes occur during health system engagement by PWUD.

**Methods:**

We propose a realist review approach due to its explanatory lens. Through preliminary exploration of literature, lived experience input, and consideration of formal theories, an explanatory model was drafted. The model describes contexts, mechanisms, and health outcomes (e.g., mental health, stable/safer drug use) involved in health system engagement. The explanatory model will be tested against the literature and iteratively refined against formal theories. A participatory lens will also be used, wherein PWUD with lived experience of health system engagement will contribute throughout all stages of the review.

**Discussion:**

We believe this is the first realist review to explore the contextual factors and underlying mechanisms of health outcomes for PWUD who participate in health system engagement. A thorough understanding of the relevant literature and theoretical underpinnings of this process will offer insights and recommendations to improve the engagement processes of PWUD.

**Supplementary Information:**

The online version contains supplementary material available at 10.1186/s13643-022-01938-z.

## Background

Health system engagement refers to the direct involvement of people with lived and living experience of a specific health issue or identity in the planning, delivery, quality improvement, research, evaluation, and advocacy relating to the health system. Common examples of engagement include membership within a quality council, provision of ad hoc feedback on new programs, and employment as an outreach or support worker or patient researcher. Community participation in health system engagement is associated with beneficial system and process transformations [1, 2]. Espousing the principles of meaningful community engagement, health systems professionals, and academics have increased their engagement of people who use or have used illegal drugs (PWUD), engendering change through group action and ground-up decision-making [3]. This engagement has facilitated the funding for and expansion of vital health services and interventions for PWUD (e.g., needle and syringe programs) [4, 5] and increased the relevance, validity, and credibility of policy and academic work, facilitating access to community-based knowledge and uptake of knowledge by key stakeholders [6].

In addition to systems-level outcomes, PWUD who engage as health system actors beyond the service recipient role also experience individual-level impacts on their health and well-being (i.e., physical, social, emotional, or spiritual benefits or harms). Through engagement, PWUD have described experiencing enhanced social and professional skills, acknowledgement and financial validation of their expertise, self-described positive changes in drug use and other behaviors, and a salubrious transformation in self-perception from a social and health service user to a service provider [7–9]. The broader patient engagement literature echoes the individual benefits of meaningful engagement [10, 11].

Unfortunately, healthcare and academic spaces may paradoxically act as risk environments wherein PWUD routinely face barriers to engagement through the imposition of structural and attitudinal obstacles to their participation [12]. PWUD have described insufficient recognition and support for their emotional and physical vulnerability, inadequate pay and lack of long-term career opportunities, and perceptions of tokenization [13–16]. Additionally, PWUD have been traditionally underserved or potentially harmed by conventional health systems and have reported mistrust towards academia and medical establishments in general [17, 18]. Studies have shown that whereas thoughtful and authentic engagement can support PWUD, misguided or inauthentic engagement may traumatize and compromise health rather than foster well-being [3, 19].

The engagement of PWUD in health system activities is a unique process that must be carefully considered rather than extrapolated from general population best practice. Harm reduction practitioners and PWUD have developed guidance to facilitate meaningful engagement, emphasizing processes that are likely to generate equitable, culturally safe, and beneficial outcomes and prevent harms [20–22]. However, there remains a range of health outcomes for PWUD involved in engagement, with several processes and contextual factors influencing this experience and little explanation provided for this variation to guide engagement practices [3, 16]. Therefore, we seek to explore the underpinnings of what engagement approaches work or do not work for PWUD, and why and under what circumstances these approaches succeed or fail in promoting well-being. Through this process, we aim to refine existing best practice recommendations for engaging PWUD, which are needed to ensure positive experiences and beneficial outcomes for all individuals involved and the health system as a whole. In keeping with the complex realities faced by PWUD engaged in the health system, a realist approach was chosen. The protocol for this study follows.

## Objectives and research questions

The objectives of the proposed realist review are to:

1. Examine how engaging in health system activities (e.g., health service planning and delivery, engaged scholarship, collaborative advocacy) influences the health and well-being of PWUD;
2. Develop recommendations for PWUD engagement in health system activities that support their health and well-being, minimize negative impacts, and engender health system change.

Specific research questions are the following:

1. How, why, for whom, and under what circumstances does health system engagement *improve* the health and well-being of individual PWUD?
2. How, why, for whom, and under what circumstances does health system engagement *worsen* the health and well-being of individual PWUD?

## Methods

### Study design

Realist reviews are rooted in a critical realist philosophy and subscribe to a generative model of causality, meaning to deduce an outcome from a health system intervention, one must understand the mechanisms and contexts of the intervention as it is situated within its complex overarching structure [23, 24]. We will therefore synthesize not only the positive and negative outcomes of health system engagement, but also the underpinnings of these varied outcomes, thus uncovering the “why” and “how” of health outcomes that arise from an intervention.

We will use the key steps of a realist review identified by Pawson et al. [23] and informed by the Realist and Meta-narrative Evidence Syntheses: Evolving Standards (RAMESES) project [25, 26]. This iterative and non-linear approach includes (i) clarifying scope (i.e., identifying the purpose of the review and articulating key theories), (ii) searching for evidence, (iii) appraising the studies and extracting data, (iv) synthesizing evidence and drawing conclusions, and (v) disseminating, implementing, and evaluating recommendations [23] (see Additional file 1 for PRISMA-P diagram).

### Clarifying scope

**Fig. 1:**
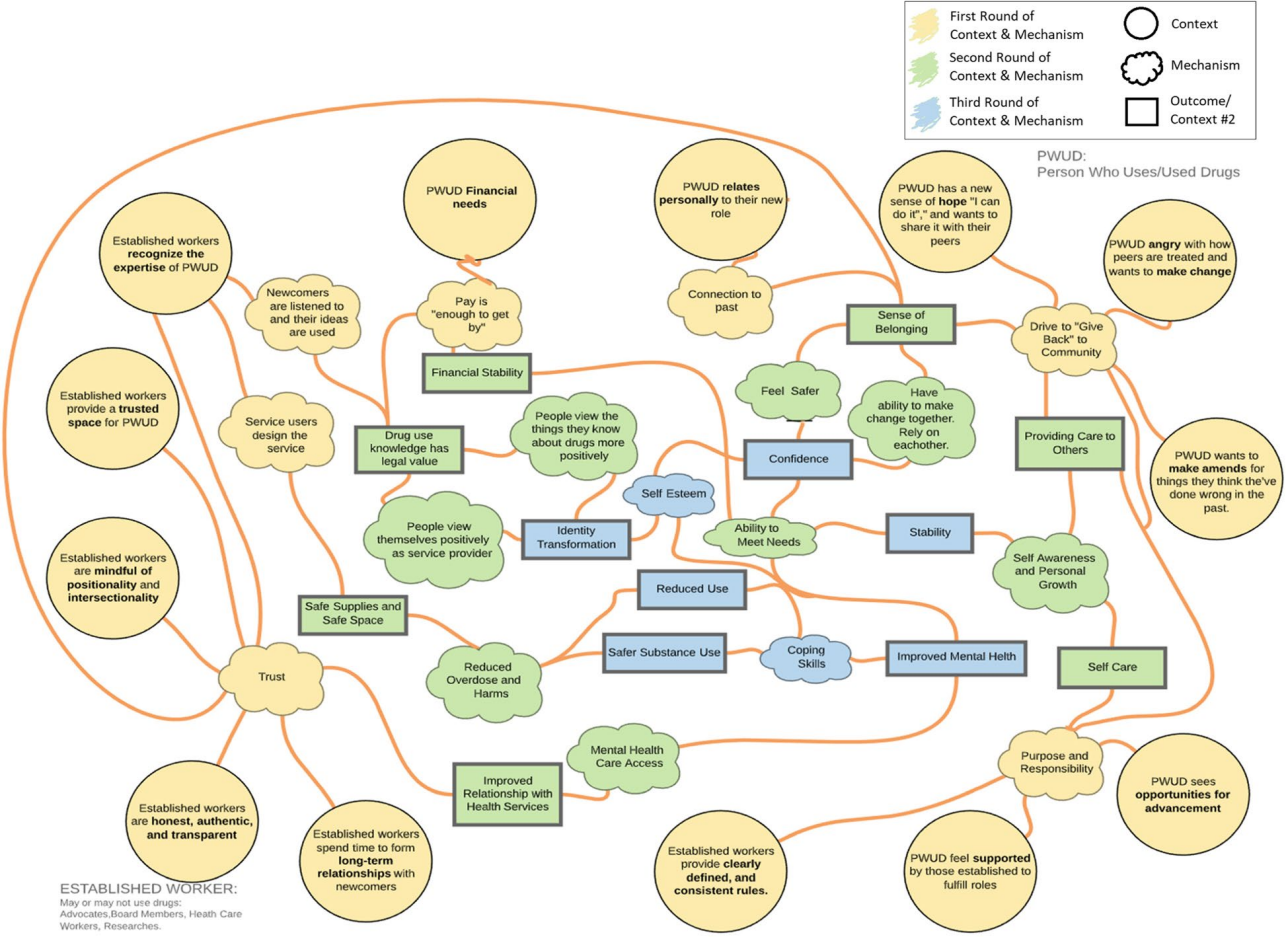
Program theory

Early exploration, adjudication, and refinement of program theory offer a sketch of the terrain we wish to investigate (Fig. 1). Program theory describes how and why specific interventions are expected to work; in this case, it provides a broad explanatory model of how health system engagement (the “program”) is thought to achieve its impacts. Our team includes clinicians, academics, and PWUD. As a team, we identified and refined our questions of interest through iterative discussions. PWUD team members then described the possible contexts and mechanisms at play during health system engagement, and we complemented this discussion with a preliminary search of the literature for key theoretical and empirical publications that reflected this lived experience. Using these team discussions and literature sources we developed a draft visual representation of program theory through which to frame the subsequent steps of the review.

We anticipated that mechanisms within health system engagement activities would vary considerably depending on intersecting influences, and as such, we approached this review with no *a priori* hypothesis. However, consistent with realist review methods, we explored the potential explanatory role of candidate formal theories in describing our developing program theory [23, 27]:

- *Resilience theory* describes the process of transforming adversity into a growth, in particular by finding strength through situational, philosophical, relational, and ego-related constructs [28].
- *Relationship-centered care* emphasizes the relational aspects of care and the healing processes. In relationship with others, we identify and embody concepts which lead to the development of intrinsic motivation [29].
- *Relational empowerment theory* is a component of overall empowerment theory. It involves power being generated in the space of interpersonal exchanges. In addition, a component of relational-empowerment theory speaks to the confidence gained from solidarity in group membership, information sharing, and mutual obligation [30].
- *Subjugated knowledge theory* explores the relationship between power and knowledge. Foucault claims that some knowledges have been invalidated and that one can find power and health through reclaiming invalidated knowledge [31].
- *Social movement theory* explores the motivators of change, the framing of change messages, and to a lesser extent enhanced personal identity through aligning self with a larger social movement [32].
- *Assumptive loss theory* examines how trauma disorients and destabilizes one’s core beliefs and what interventions can be used to heal from this loss [33]*.*

Realist review is an iterative process of engaging with the literature and the mechanics operating behind the apparent outcomes [34]. As such, we expect to further negotiate and potentially narrow our area of focus as priorities become apparent.

### Searching for evidence

Realist reviews comprise complementary search strategies and several iterative yet progressive steps that are guided by emergent criteria eventually leading to a refinement of program theory [23, 35]. Consistent with iterative search strategies articulated by realist methods experts [35], we conducted an exploratory background search [23] using a single database (i.e., MEDLINE) to gain perspective on the literature, using search terms derived from the following:

- Population: people who use drugs (i.e., any unsanctioned use of opioids/stimulants/illegal substances, *specifically excluding* exclusive use of cannabis/alcohol/tobacco)
- Process: activities related to health system engagement in the form of planning, delivery, or research (i.e., health service planning, quality improvement, evaluation, research, collaborative advocacy, whether as an internal actor (e.g., front-line outreach/peer support/clinician) or an external actor (e.g., patient advisor), *specifically excluding* healthcare seeking or receiving as a patient/client
- Outcome: individual health and well-being (i.e., any personal physical/emotional/social/spiritual health impacts, *specifically excluding* collective/community health impacts)

We will use the articles retrieved in this first search to clarify the review scope, refine inclusion and exclusion criteria, and test potential theoretical fit [23, 35]. Two reviewers will test and refine document inclusion and exclusion criteria on initial search results until > 80% agreement is reached [36]. We will subsequently apply pre-specified search terms and modifiers, with assistance from a university librarian, and conduct a more in-depth search of multiple databases (i.e., MEDLINE, CINAHL, Embase, PsycINFO) to identify further articles meeting inclusion criteria.

Due to an expectation of potentially few search results, and the need to explore contextual factors, we will employ secondary cluster searching [37]. Cluster searching involves targeted searches and purposive sampling of key journals, including unpublished documents, and grey literature, and contacting authors and researchers for further information regarding a project. We may also search the non-PWUD literature to clarify emerging mechanisms of engagement as they play out for diverse populations and settings. By utilizing multiple search strategies, we will enhance the contextual richness of our findings and interpretation of the data.

Two reviewers will independently screen and select documents that fit our inclusion criteria and study objective, beginning with titles and abstracts and followed by full text review for selected abstracts. Where consensus cannot be achieved after discussion, a third reviewer will determine document eligibility. We will maintain a Covidence software-supported record of the screening process, including a rationale for inclusion and exclusion decisions [38]. To fully investigate program theory, we will not restrict by study type.

### Appraising studies and extracting data

We will appraise each study based on its relevance (i.e., whether it addresses the theory under review) and its rigor (i.e., whether the inferences made within the study contribute to a test of its theory) in relation to our initial program theory and study objectives [23, 35]. Data appraisal will occur simultaneously but independently of data abstraction by two independent reviewers. Disagreements will be resolved through discussion and involvement of a third reviewer if necessary.

Selected articles will be independently extracted by two reviewers, using standardized data extraction forms and critical appraisal checklists adapted to study design [23, 39]. Data extraction will focus on context-mechanism outcome configurations relevant to our initial program theory—specifically, the context in which the engagement took place, its interaction with mechanism of actions as suggested by the authors or inferred from the study, and the impact on individual-level outcomes. Extracted information will include the study population and demographics, study type and method, study setting including country and community context, role of PWUD, recruitment and selection, training and support, reimbursement and incentives, and drop-out rates. We will engage in iterative discussion with all research team members, including PWUD with lived experience of health system engagement, to ensure consensus on extracted data. Though critical appraisal has limited value in realist reviews, this step is included to fully describe the literature; with the exception of major methodological flaws compromising a manuscript’s findings (wherein caution would be exercised in applying extracted configurations to program theory), the critical appraisal process will not otherwise influence the development of context-mechanism outcome configurations. Data saturation will be assessed after each round of extraction and we will stop searching when more literature is unlikely to add new knowledge to the review [23].

### Synthesizing evidence and drawing conclusions

**Fig. 2:**
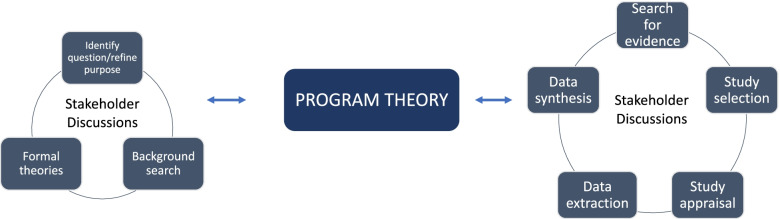
Data extraction and synthesis

Using an iterative approach wherein researchers interact in an alternating fashion with the data and related theory (Fig. 2), we will synthesize abstracted context-mechanism outcome configuration data to identify demi-regularities (emerging patterns) and construct and refine an overarching context-mechanism-outcome framework for PWUD engagement, i.e., what engagement processes work (or do not work) for whom and in what circumstances, how they work, and what are the resulting individual-level outcomes [23]. We will use a variety of source types to refine and test theories, and support or refute inferences about mechanisms. We will actively seek “negative” or contradictory cases to examine the impact of intersecting contextual influences on the engagement process [40].

### Disseminating, implementing, and evaluating recommendations

Dissemination and knowledge translation activities will include the development of best practice and evaluation recommendations for involving PWUD in health system activities, policy briefs, and scientific presentations and publications, as well as identification of key knowledge gaps. Findings from the review will be co-messaged by PWUD wherever feasible, using a variety of formats including information sessions hosted by community organizations, plain language summaries, infographics, and virtual presentations.

### Team composition

We propose a participatory lens of key stakeholders to carry out the proposed review [15]. Academics, clinicians, students, and PWUD with lived experience of health system engagement will contribute to the work across all stages of the review, including refinement of review questions and purpose, development of the search strategy, selection of literature, data abstraction, analysis, and dissemination. Additionally, although Indigenous peoples are not the main focus of our study, a larger proportion of the PWUD community in Canada identify as Indigenous compared to the general population [41]. More than half of the PWUD members of the project team identify as Indigenous. Their presence will help to ensure that the study design, data collection, interpretation, and dissemination directly reflect the priorities of Indigenous and non-Indigenous PWUD alike and are conducted in a culturally safe manner [42].

We also recognize that any scholarship on PWUD requires close attention to researcher positionality and reflexivity. Researchers will reflect on their own identities and roles, consider how these influence data collection, analysis, and interpretation, and take measures to address potential personal and professional bias [43].

## Discussion

To the best of our knowledge, this is the first review to explore the health impacts of health system engagement on PWUD. Realism allows us to understand how and why some engagement activities benefit PWUD whereas others can cause harm. Our team commits to the active involvement of PWUD throughout all stages this review to (1) ensure that emerging theory reflects their experience rather than the perception of external researchers and (2) co-develop recommendations for healthy engagement strategies. We will ensure that PWUD team members receive support in scheduling and attending meetings with an acceptable time, location, and frequency, that they be given the opportunity to participate inasmuch as they desire and find feasible, and that they are fairly and financially compensated for their expertise (VD is an author of this manuscript and KD and MK are acknowledged for their expertise). We further commit to following up promptly with PWUD team members on issues arising during the review, actioning recommendations that emerge from the review in our own scholarly work, and directly advocating for uptake of recommendations by review stakeholders (e.g., health authorities, academic institutions, addiction, and mental health organizations).

## Limitations

As with all reviews, this review is at risk of publication bias; this will be addressed using multiple databases and secondary cluster searching. Researcher bias will be mitigated using a two-person independent screening, appraisal, and abstraction approach, along with the iterative involvement of multiple team members from a variety of backgrounds during theory development. Moreover, unlike systematic reviews, realist reviews are deliberately context-specific and may not be generalizable to all PWUD in all settings; we are approaching this review with the understanding that the primary knowledge gap is the “how” and “why” of engagement’s health impacts, and will search for pattern variation according to the people and settings involved as we refine emerging theory. We acknowledge that our work reflects extant public knowledge and that our findings can and should stimulate further study of the complexity involved in healthy engagement of PWUD.

## Supplementary Information

**Additional file 1.**

## Data Availability

The datasets used and/or analyzed during the current study are available from the corresponding author on reasonable request.
