# Supplementary material for: Impact of health system engagement on the health and well-being of people who use drugs: a realist review protocol
Source: Syst Rev. 2022 Apr 13;11:66. doi: 10.1186/s13643-022-01938-z (PMC9008896; doi:10.1186/s13643-022-01938-z)
Supplement: Supplementary file 1 — Additional file 1. [file 13643_2022_1938_MOESM1_ESM.docx]

**PRISMA-P 2015 Checklist**

# This checklist has been adapted for use with systematic review protocol submissions to BioMed Central journals from Table 3 in Moher D et al: Preferred reporting items for systematic review and meta-analysis protocols (PRISMA-P) 2015 statement. *Systematic Reviews* 2015 4:1

# An Editorial from the Editors-in-Chief of *Systematic Reviews* details why this checklist was adapted - Moher D, Stewart L & Shekelle P: Implementing PRISMA-P: recommendations for prospective authors. *Systematic Reviews* 2016 5:15

| **Section/topic** | **#** | **Checklist item** | **Information reported** | | **Line number(s)** |
| --- | --- | --- | --- | --- | --- |
|  |  |  | **Yes** | **No** |  |
| **ADMINISTRATIVE INFORMATION** | | | | | |
| **Title** | | | | | |
| Identification | 1a | Identify the report as a protocol of a systematic review | ☒ | ☐ | page 2, line 17 |
| Update | 1b | If the protocol is for an update of a previous systematic review, identify as such | ☐ | ☒ | N/A |
| **Registration** | 2 | If registered, provide the name of the registry (e.g., PROSPERO) and registration number in the Abstract | ☐ | ☒ | N/A (realist review) |
| **Authors** | | | | | |
| Contact | 3a | Provide name, institutional affiliation, and e-mail address of all protocol authors; provide physical mailing address of corresponding author | ☒ | ☐ | page 1, lines 4-36 |
| Contributions | 3b | Describe contributions of protocol authors and identify the guarantor of the review | ☒ | ☐ | page 11, lines 19-28 |
| **Amendments** | 4 | If the protocol represents an amendment of a previously completed or published protocol, identify as such and list changes; otherwise, state plan for documenting important protocol amendments | ☐ | ☒ | N/A |
| **Support** | | | | | |
| Sources | 5a | Indicate sources of financial or other support for the review | ☒ | ☐ | page 11, lines 15-17 |
| Sponsor | 5b | Provide name for the review funder and/or sponsor | ☒ | ☐ | page 11, lines 15-17 |
| Role of sponsor/funder | 5c | Describe roles of funder(s), sponsor(s), and/or institution(s), if any, in developing the protocol | ☒ | ☐ | page 11, lines 15-17 |
| **INTRODUCTION** | | | | | |
| **Rationale** | 6 | Describe the rationale for the review in the context of what is already known | ☒ | ☐ | page 4, lines 4-17 |
| **Objectives** | 7 | Provide an explicit statement of the question(s) the review will address with reference to participants, interventions, comparators, and outcomes (PICO) | ☒ | ☐ | page 4, lines 19—29 and page 5, lines 1-4 |
| **METHODS** | | | | | |
| **Eligibility criteria** | 8 | Specify the study characteristics (e.g., PICO, study design, setting, time frame) and report characteristics (e.g., years considered, language, publication status) to be used as criteria for eligibility for the review | ☒ | ☐ | page 5, lines 16-20 and page 6, lines 22-28 and page 7, lines 1-3 |
| **Information sources** | 9 | Describe all intended information sources (e.g., electronic databases, contact with study authors, trial registers, or other grey literature sources) with planned dates of coverage | ☒ | ☐ | page 7, lines 9-17 |
| **Search strategy** | 10 | Present draft of search strategy to be used for at least one electronic database, including planned limits, such that it could be repeated | ☒ | ☐ | See OVID MEDLINE draft search strategy below on page 5 of this document |
| ***STUDY RECORDS*** | | | | | |
| Data management | 11a | Describe the mechanism(s) that will be used to manage records and data throughout the review | ☒ | ☐ | page 7, lines 22-24, page 8, lines 6-8 |
| Selection process | 11b | State the process that will be used for selecting studies (e.g., two independent reviewers) through each phase of the review (i.e., screening, eligibility, and inclusion in meta-analysis) | ☒ | ☐ | page 7, lines 18-24 |
| Data collection process | 11c | Describe planned method of extracting data from reports (e.g., piloting forms, done independently, in duplicate), any processes for obtaining and confirming data from investigators | ☒ | ☐ | page 8, lines 6-19 |
| **Data items** | 12 | List and define all variables for which data will be sought (e.g., PICO items, funding sources), any pre-planned data assumptions and simplifications | ☒ | ☐ | page 8, lines 8-15 |
| **Outcomes and prioritization** | 13 | List and define all outcomes for which data will be sought, including prioritization of main and additional outcomes, with rationale | ☒ | ☐ | page 8, lines 25-27 |
| **Risk of bias in individual studies** | 14 | Describe anticipated methods for assessing risk of bias of individual studies, including whether this will be done at the outcome or study level, or both; state how this information will be used in data synthesis | ☐ | ☒ | According to Pawson et al. realist reviews explore relevance and rigour, not bias. However, study appraisal is described page 7, lines 26-28 and page 8, lines 1-4 |
| ***DATA*** | | | | | |
| **Synthesis** | 15a | Describe criteria under which study data will be quantitatively synthesized | ☐ | ☒ | N/A (quant synthesis not appropriate) |
|  | 15b | If data are appropriate for quantitative synthesis, describe planned summary measures, methods of handling data, and methods of combining data from studies, including any planned exploration of consistency (e.g., *I* ^2^, Kendall’s tau) | ☐ | ☒ | N/A (quant synthesis not appropriate) |
|  | 15c | Describe any proposed additional analyses (e.g., sensitivity or subgroup analyses, meta-regression) | ☐ | ☒ | N/A (quant synthesis not appropriate) |
|  | 15d | If quantitative synthesis is not appropriate, describe the type of summary planned | ☒ | ☐ | page 8, lines 21-27 |
| **Meta-bias(es)** | 16 | Specify any planned assessment of meta-bias(es) (e.g., publication bias across studies, selective reporting within studies) | ☐ | ☒ | N/A (realist review) |
| **Confidence in cumulative evidence** | 17 | Describe how the strength of the body of evidence will be assessed (e.g., GRADE) | ☐ | ☒ | N/A (realist review) |

| **#** | **Search Statement** | **Results** |
| --- | --- | --- |
| 1 | *"Drug Users"/ or (PWID or PWUD or "people who use drugs" or "people who inject drugs" or IDU or IDUs or "intravenous drug user*" or "drug abus*" or "intravenous substance abuse*" or (addict* adj3 (intravenous or inject*))).ti,kw. or *Substance Abuse, Intravenous/ | 19261 |
| 2 | alcoholism/ or alcohol abuse*.mp. or alcoholics/ or tobacco abuse*.mp. [mp=title, abstract, original title, name of substance word, subject heading word, floating sub-heading word, keyword heading word, organism supplementary concept word, protocol supplementary concept word, rare disease supplementary concept word, unique identifier, synonyms] | 84898 |
| 3 | 1 and 2 | 979 |
| 4 | 1 not (2 not 3) [Drug uses - tobacco and alcohol removed] | 19261 |
| 5 | exp Outcome Assessment, Health Care/ or health [outcomes.mp](http://outcomes.mp/). | 1176903 |
| 6 | Program [evaluation.mp](http://evaluation.mp/). or exp Program Evaluation/ | 79873 |
| 7 | ("natural helper*" or "expert patient*" or (health adj3 (buddy or buddies)) or consumer case management or assertive community treatment* or consumer-provider* or "patient activation" or "patient led" or "user led" or "user run" or "patient run" or ((patient* and (research or health system* or nonclinical* or "non clinical*")) adj3 (advoca* or engag* or involv*)) or (patient* adj3 (co-design or govern* or collaborat* or partner* or educator*)) or (("Service user*" or consumer* or Peer*) adj3 (researcher* or worker* or educator* or provider* or based or led or support* or nonclinical* or "non-clinical*")) or ((consumer* or peer* or "service user") adj5 (leadership skills or educating or education or learning or training or (research adj5 (involv* or engag*)) or professional development or capacity building or reimbursement* or incentive* or honorarium* or compensation or conference attendance or stipend* or govern* or co-design* or advisory board*))).mp. | 61165 |
| 8 | exp Community-Based Participatory Research/ or Consumer Participation/ or Stakeholder Participation/ or exp Community Participation/ or ((consumer* or stakeholder* or patient* or community) adj2 (activation or activated or oriented or driven or engage* or empower* or framework* or approach* or guidance or guide or implement* or involvement or participat* or partner* or research* or tool or toolkit* or technique*)).mp. | 185064 |
| 9 | 7 or 8 | 233043 |
| 10 | 4 and 9 | 518 |
| 11 | 5 or 6 [assessment and evaluation set] | 1247979 |
| 12 | 10 and 11 | 83 |
